# Arthroscopic reduction and internal fixation for fracture of the posterior process of the talus (Shepherd’s fracture): a case report

**DOI:** 10.1186/s13256-024-04652-7

**Published:** 2024-07-30

**Authors:** Branislav Krivokapic, Pieter DHooghe, Nikola Bogosavljevic, Danilo Jeremic, Nina Rajović

**Affiliations:** 1 Institute for Orthopaedic Surgery “Banjica”, Mihajla Avramovica 28, 11000 Belgrade, Serbia; 2 Faculty of Medicine, University of Belgrade, Dr Subotica 8, 11000 Belgrade, Serbia; 3 Aspetar Orthopedic and Sports Medicine Hospital, Aspire Zone, Sport City Street 1, PoBox 29222, Doha, Qatar

**Keywords:** Fractured posterior process of the talus, Shepherd fracture, Posterior ankle arthroscopy

## Abstract

**Introduction:**

Fracture of the lateral tubercle of the posterior process of the talus (Shepherd fracture) is an uncommon injury seen in sport. It is secondary either to indirect trauma on the plantarflexed foot or to high-impact direct trauma. The fracture can be missed with conventional X-rays and therefore advanced imaging methods such as CT scans are usually warranted for management planning. There is a low threshold towards surgical management in the displaced or comminuted case as the delayed functional outcome with conservative treatment is frequently sub-optimal with long-term pain, degenerative changes and non-union. In this regard, recent years saw an increasing interest in the role of minimally invasive approaches for Shepherd´s fracture treatment, such as arthroscopic reduction and internal fixation (ARIF).

**Case report:**

We present a case of a 27-year-old white male professional football player from Serbia who had Shepard fracture and successfully managed with arthroscopic osteosynthesis. The technical approach is detailed with posterior ankle arthroscopy offering the advantages of a minimally invasive approach with low morbidity and a rapid return to regular sporting activities.

**Conclusion:**

The utilization of the 2-port arthroscopic approach this method enables the direct observation of the articular surface along with the corresponding fracture lines, thereby affording the surgeon the chance to achieve accurate reduction via a minimally invasive soft tissue aperture. We advocate that Arthroscopic reduction and internal fixation (ARIF) is a reliable method for the fixation of Shepherd's fracture in the hands of experienced ankle arthroscopists.

## Introduction

Talus fractures constitute only 0.3% of all fractures and most commonly involve the talar head and/or the neck [1]. A fracture of the posterior process of the talus (PPT) is particularly rare [2], representing a mere 3–5% of all foot fractures. [3]. The PPT fracture was first reported by Shepherd in 1882 [4] with a case series reported by Cedell in 1974 [5]. Fractures of the tubercles are therefore known as the lateral tubercle fracture (Shepherd fracture), which is more frequent, and the medial tubercle fracture (Cedell fracture).

### Mechanism of injury

Two discrete mechanisms and directional forces have been proposed to explain the different injury patterns. One is a forced hyper plantarflexion with inversion of the foot, resulting in direct compression of the posterior talus between the posterior tibial rim and the dorsal rim of the posterior facet of the calcaneum. Another is by the force of the posterior talofibular ligament causing an avulsion fracture of the lateral tubercle during a hyper dorsiflexion and inversion movement [6]. In the description by Cedell, a fracture avulsion of the posteromedial tubercle is a consequence of forced pronation and dorsiflexion of the foot [7]. The rarer type of injury is the effect of direct trauma on to the posteromedial facet. In this event, there is a high energy impingement onto the sustentaculum tali when the foot is forcibly dorsiflexed whilst supinated.

### Diagnostic methods

In more than 40% of cases, a fracture of the posterior process of the talus is overlooked at clinical presentation. Although swelling and pain are usually evident on the posterior side of the ankle, there is pain on palpation of the projection of the posterior talar process as well as a positive apprehension test with flexion of the FHL tendon of the great toe (the FHL sign).

The Cedell-type posteromedial process talar fracture may be mistaken for a simple ankle sprain where posteromedial pain is frequently underestimated [2, 8]. Similarly, a Shepherd-type fracture may be mistaken for pain from an os trigonum, a secondary ossification center which can fail to fuse to the talar posterolateral process. A Shepherd fracture can equally be misdiagnosed as an ankle sprain although with such fractures pain is typically reproduced on movement of the subtalar joint and with elicitation of the FHL sign.

Standard diagnostic X-ray imaging of the ankle is performed with anteroposterior, mortise and lateral views [2]. When insufficient, 2 additional 45° and 70° external rotation oblique views (as advocated by Ebraheim) should be obtained [9]. Since there is a 40% missed diagnosis rate with initial radiography [10, 11], and given the need for timely diagnosis in order to achieve optimal functional outcome, suspicion of such an injury usually warrants additional imaging with either CT or MRI [12]. A CT scan identifies the fracture displacement as well as its comminution and the positional relationship of the fracture fragments, whereas an MRI defines more soft-tissue detail including the status of the FHL tendon [13].

To date, different open surgical approaches have been practiced in order to operatively reduce and fix posterior talar fractures. However, a considerable risk of complications and a poor prognosis were associated with these surgical methods [14].

Recent years saw an increasing interest in the role of minimally invasive approaches for PPT treatment, such as ARIF. So far, arthroscopy has been routinely practiced for diagnostic evaluation and treatment of various talar pathologies, however there is a paucity of data regarding the usage of this technique. By reducing trauma to the soft-tissue envelope, ARIF enables direct observation of the therapeutic process, anatomic reduction of the articular surfaces, and maintenance of the osseous blood supply [15].

Due to its relative rarity, we therefore present a case of a Shepherd's fracture managed solely with ARIF, highlighting the use of a minimally invasive approach for intra-operative decision-making.

## Case report

### Presentation and diagnosis

**Fig. 1:**
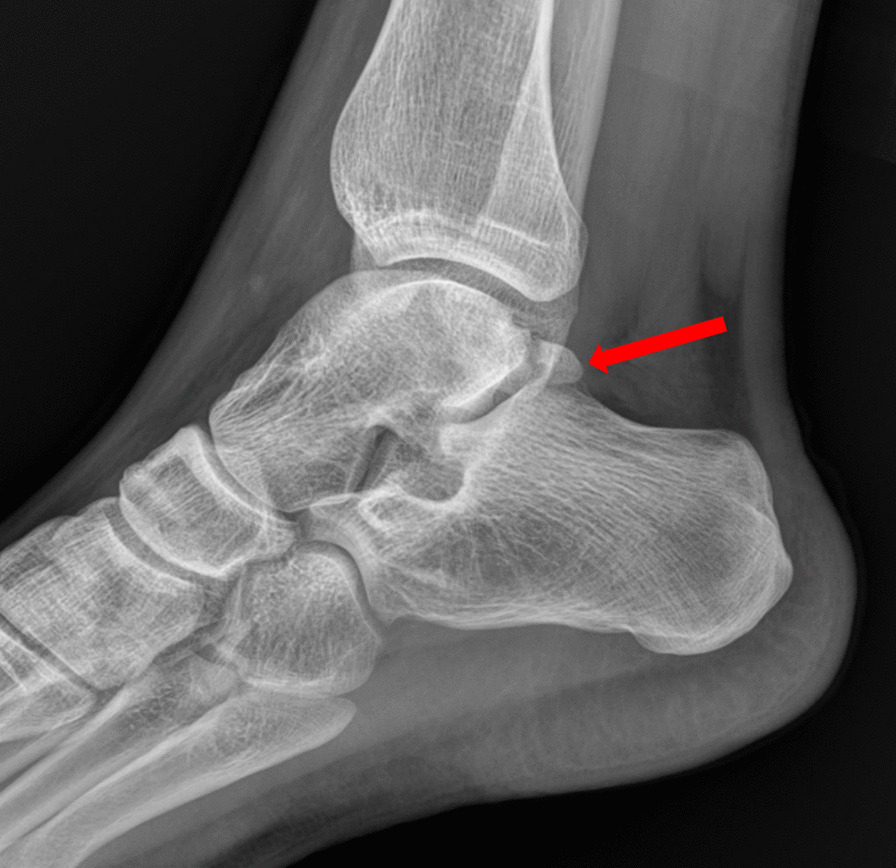
Lateral X-ray showing a posterior lateral tubercle fracture of the posterior process of the talus (Shepherd fracture). The red arrow points to the fracture site

**Fig. 2:**
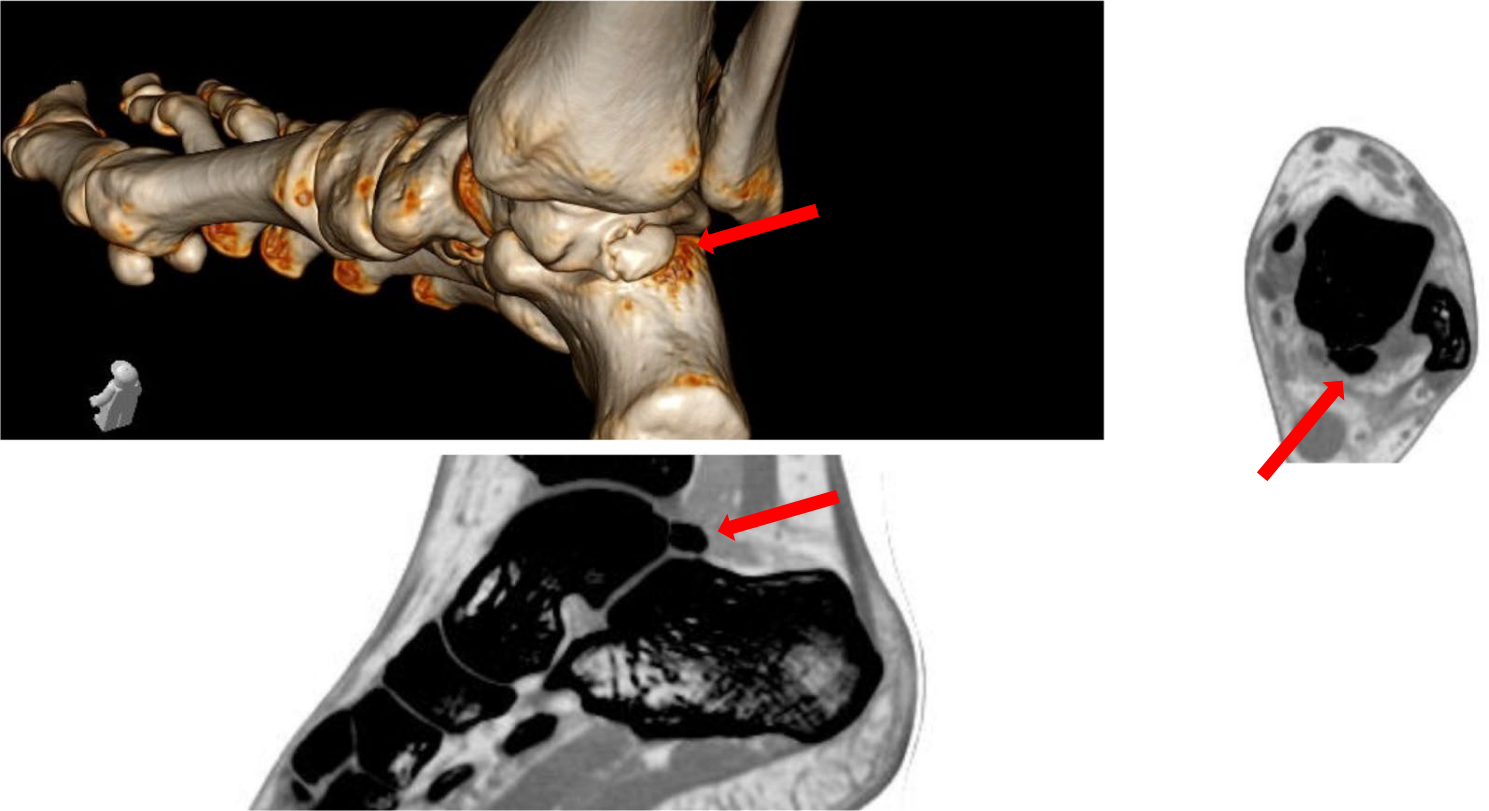
Preoperative volume rendering (VRT) CT and multiplanar reconstructions permitting better visualization of the fracture, the degree of displacement and comminution of the posterior process. Based on these factors surgical decision is made either for osteosynthesis or fragment excision. The red arrow points to the fracture site

A case is presented of a 27-year-old white male professional footballer from Serbia with an acute right ankle injury. The patient could not recall the precise mechanism of injury but volunteered that his foot was likely plantarflexed when landing. On physical examination there was visible swelling, ecchymosis and tenderness to palpation of the posterior ankle with a positive FHL test. In Fig. 1 an X-ray with clear evidence of a Shepherd fracture of the lateral tubercle of the PPT is shown. In Fig. 2 the preoperative CT scan of the patient including axial and sagittal multiplanar reconstructions and a volume-rendered image, the latter better showing the fracture, its displacement and comminution, is shown. The CT showed significant displacement of a large (17 mm × 10 mm) fragment. Decision was made to proceed to a posterior hindfoot arthroscopy under general anaesthesia with the patient in the prone position and using a thigh tourniquet (280 mmHg) 5 days after injury.

### Surgical fixation

**Fig. 3:**
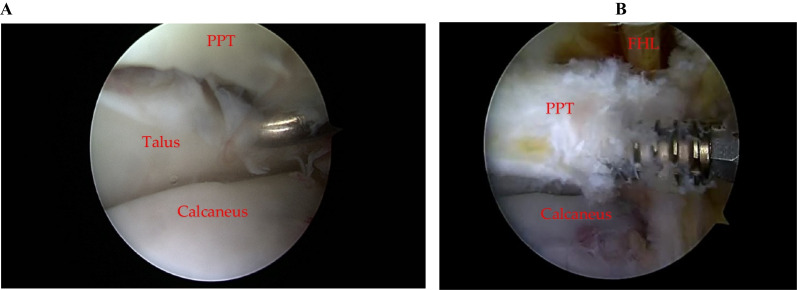
Arthroscopic appearance of the osteochondral fragment PPT (Left) and screw fixation of the fracture against the posterior talus (Right)

**Fig. 4:**
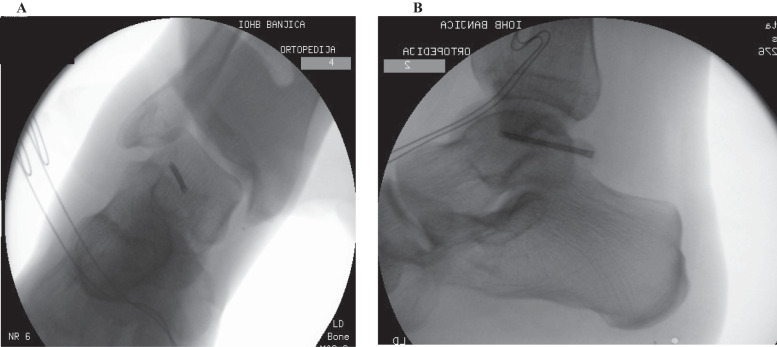
Intraoperative imaging confirming fragment reduction (**A** AP, **B** Lateral)

The procedure was performed with antibiotic cover (1 g IV Cefazolin) on induction of anaesthesia and with thromboprophylaxis (Nadroparin 1 mL S/C until the 21st postoperative day). Arthroscopic fixation was conducted via posterolateral and posteromedial portals established at the level of the top of the lateral malleolus medially and laterally alongside the Achilles tendon. A 4 mm. arthroscope was inserted via the posterolateral portal with an arthroscopic shaver inserted via the posteromedial portal for an ankle capsulotomy. Following initial débridement, the broken fragment of the posterolateral process of the talus was identified, cleaned and reduced (Fig. 3A). After determination of the fracture size, it is fixated (Fig. 3B) using a fully threaded headless 2.5 mm. titanium compression screw (Arthrex, Naples FL) using the drill sleeve to maintain reduction. In Fig. 4 the intraoperative appearance checked by C-arm fluoroscopy is shown. The total tourniquet time during the surgery was 50 minutes.

### Postoperative monitoring

**Fig. 5:**
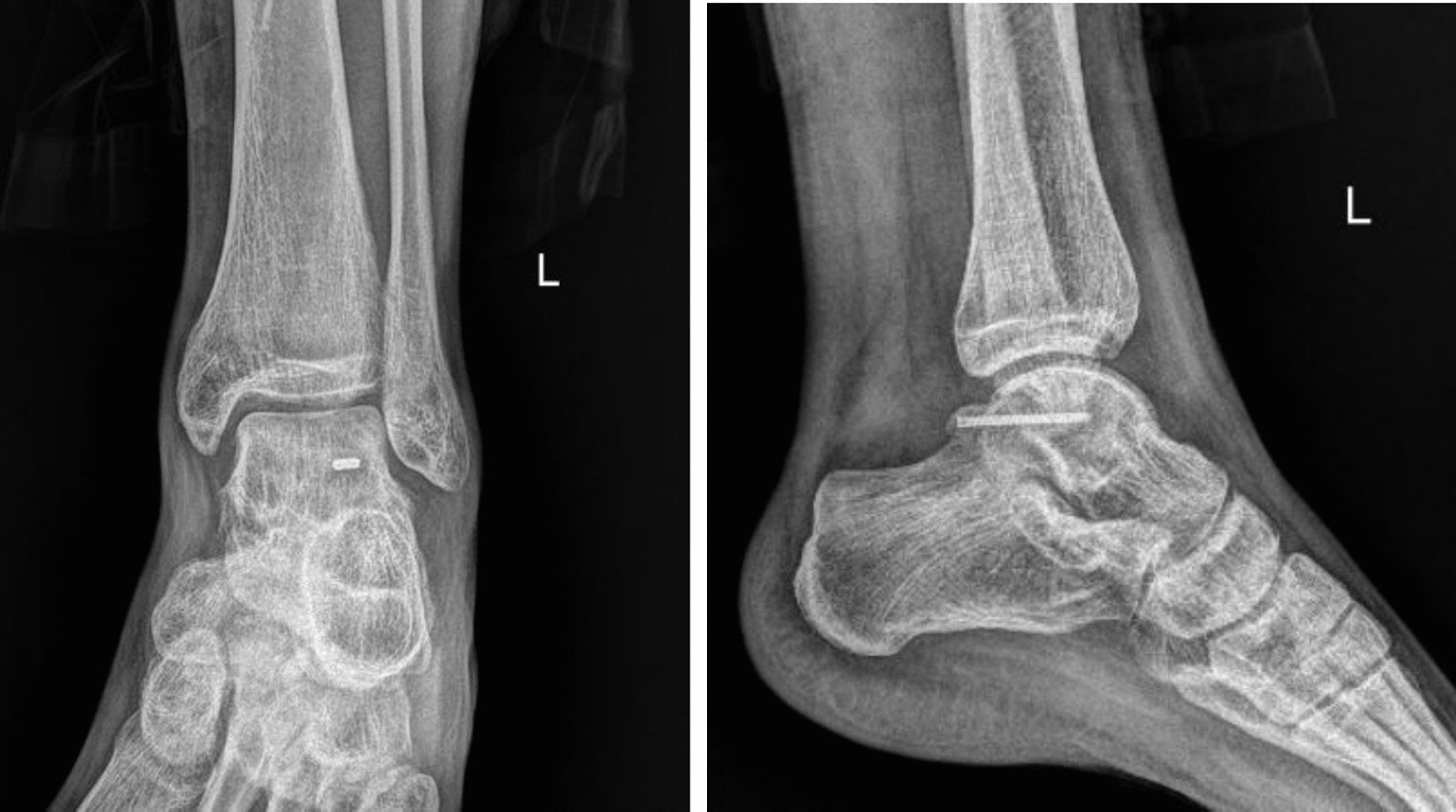
X-ray 6 weeks after surgery (AP, lateral)

The patient was discharged the day after surgery in a non-weightbearing walking boot. At 2 weeks the wound was examined with removal of sutures, commencing physical therapy by taking the ankle through a passive range of motion and permitting partial weightbearing in the walking boot for 6 weeks. After this time, there was a painless full range of ankle motion and a negative Flexor halucis longus (FHL) test. A repeat X-ray was performed showing evidence of fracture union (Fig. 5). The patient was permitted to fully weight bear and approved for return to his regular sporting activity.

## Discussion

This report presented a case of a Shepherd fracture of the lateral tubercle of the posterior process of the talus which was successfully managed by arthroscopic fixation. There are some inherent general features about the talus which may contribute to significant functional problems after injury including its complex anatomy, its involvement in multiple joints (tibiofibular, talocalcaneal and talonavicular), the large surface area that is covered by cartilage and its relatively poor vascularity [16, 17]. The functional outcome and the ability to return to the normal pre-injury level of activity can, however, be impaired in a significant number of cases. In this regard, Engelmann *et al*. have suggested that in those patients initially managed non-operatively (or where operative fixation was delayed), that there is a higher rate of complications which include chronic painful non-union, post-traumatic osteoarthritis and posterior ankle impingement. These patients have also been shown to have a greater need for secondary arthrodesis [1].

Data comparing an operative and a conservative approach are retrospective and non-randomized, although most of the literature suggests a better outcome and higher ultimate patient satisfaction amongst the cases where operation was performed in a timely manner [9, 18]. Studies available are not strictly comparable because they consist of heterogeneous reports on small numbers of cases in which the indication for surgery is sometimes ambiguous. Despite the fact that in some series those fractures typically treated nonoperatively tend to be less severe, the preponderance of worse outcomes when compared with open reduction and internal fixation (ORIF) would support surgical management. Following 410 patients managed either with open or endoscopic treatment for posterior ankle impingement Zwiers *et al.* [19], demonstrated a faster recovery and a more rapid return to sporting activity in the arthroscopic group when compared with the open surgery group (11.3 weeks versus 16 weeks, respectively) as well as a lower overall rate of complications (7.2% versus 15.9%, respectively). Given this background, surgical management is recommended for cases where there is displacement (even if minimal), where the fracture extends into the body of the talus or where there is articular involvement. The optimal treatment remains uncertain and largely depends upon the fracture pattern where a large displaced bony fragment particularly involving the subtalar joint would mandate surgical intervention. Whichever surgical management is opted for (fragment excision or osteosynthesis), there is the choice of an open or an arthroscopic approach [20, 21]. In open surgery, either a posterolateral or a posteromedial approach can be used for the different tubercle fractures, however, this type of surgery leads to significant soft-tissue trauma because of the depth of dissection required for access to the PPT and increases the risk of significant postoperative sepsis or even a chronic, non-healing wound [2, 17]. Much of this morbidity may be obviated with a minimally invasive method [22–24], although it is accepted that percutaneous screw fixation can be difficult to perform via this approach. Generally, a 2-portal hindfoot arthroscopy provides excellent access to the posterior ankle and the subtalar joint as well as to the extra-articular structures. Nowadays, clinicians should aim where possible to opt for ankle arthroscopy which permits the identification of loose bodies and injuries to the cartilage and which can reveal transchondral defects and the quality of residual subchondral bone not obvious on X-ray. The standard portals used can readily be interchanged for alternate viewing and instrumentation. Accurate arthroscopic fragment placement ensures good longer-term functional biomechanics with the arthroscopic approach avoiding the soft-tissue trauma that normally accompanies open surgery and fragment mobilization. Avoidance of this external trauma can diminish the likelihood of serious complications including infection, malunion, avascular necrosis and chronic arthritis. While ARIF is considered a more advanced surgical procedure than ORIF, it is important to note that there are potential problems such as superficial peroneal neuritis and damage to the neurovascular bundle. Ankle arthroscopy identified a notable prevalence of OCL (osteochondral lesions), ligamentous injuries, and loose bodies in individuals with ankle fractures. The duration of the surgical procedure for ARIF may be extended, particularly for surgeons with less expertise, and the process of acquiring proficiency may take a considerable amount of time [25, 26]. Nevertheless, when surgical knowledge advances, physicians will no longer have to be concerned about the potential occurrence of postoperative complications or prolonged surgical durations. Therefore, ankle arthroscopy may be used as a diagnostic and prognostic modality, alongside its role in the treatment of ankle fractures. This technique is advantageous for detecting and controlling irregularities inside the joint and may aid in preventing the development of osteoarthritis after trauma.

## Conclusions

CT scanning has been advised as the preferred imaging modality in case of the PPT where a fracture is suspected. ARIF represent a promising technique with minimal complications that may facilitate expedited patient mobilization. The utilization of the 2-port arthroscopic approach this method enables the direct observation of the articular surface along with the corresponding fracture lines, thereby affording the surgeon the chance to achieve accurate reduction via a minimally invasive soft tissue aperture. We advocate that ARIF is a reliable method for the fixation of Shepherd's fracture in the hands of experienced ankle arthroscopists.

## Data Availability

Not applicable.

## Abbreviations

**ARIF**: Arthroscopic reduction and internal fixation
**PPT**: Posterior process of the talus
**FHL**: Flexor halucis longus
